# The Prognostic Value of Combined Complete Blood Count and Immune Cell Profiling in Patients with Multiple Myeloma Treated with Chemotherapy Sequential Transplantation

**DOI:** 10.3390/cancers18152389

**Published:** 2026-07-24

**Authors:** Jiang Zhang, Yao Chen, Yizhi Mao, Yaoming Chen, Mengzhi Hong, Junxun Li, Juan Ouyang

**Affiliations:** Department of Laboratory Medicine, The First Affiliated Hospital of Sun Yat-Sen University, Guangzhou 510080, China; zhangj238@mail.sysu.edu.cn (J.Z.);

**Keywords:** multiple myeloma, minimal residual disease, complete blood count, immune cell profiling, autologous stem cell transplantation

## Abstract

**Background:** Multiple myeloma (MM) is an incurable hematologic malignancy, and the majority of patients eventually relapse due to persistent myeloma cells. While minimal residual disease (MRD) reflects tumor burden, the host immune system also plays a crucial role in disease control. Our study aimed to evaluate MRD, complete blood count (CBC), and immune cell profiles in MM patients treated with bortezomib/adriamycin/dexamethasone (PAD) chemotherapy followed by autologous stem cell transplantation (ASCT) to determine their prognostic value and interplay. **Objectives:** This study aims to identify markers indicative of a favorable prognosis in MM patients. **Methods:** CBC data were collected from 93 MM patients at diagnosis, prior to ASCT, and 3 months post-ASCT. Immune cell profiles were assessed via flow cytometry using fresh peripheral blood samples from a subset of 33 prior to ASCT and 3 months post-ASCT. We subsequently investigated the associations between MRD status and prognosis, the predictive value of CBC, and the longitudinal changes in immune cell profiles and their correlation with clinical outcomes. **Results:** An increased frequency of negative immunomodulatory cell subsets and activated T lymphocytes prior to ASCT were associated with a poor prognosis. Conversely, lower levels of exhausted T lymphocytes, alongside higher levels of functional T cells and marginal zone B cells post-ASCT, predicted a favorable prognosis. **Conclusions:** Our study demonstrates that pre-ASCT immune status, post-ASCT immune reconstitution, and MRD status at 3 months post-ASCT are closely associated with clinical outcomes in MM patients. These findings underscore the critical importance of regular immune monitoring in the comprehensive management of MM.

## Simple Summary

Multiple myeloma is a hematologic malignancy characterized by high relapse rates, rendering it largely incurable. Although clinical monitoring primarily focuses on minimal residual disease (MRD), the host immune system plays an indispensable role in disease control. In this study, we evaluated longitudinal immune cell dynamics in peripheral blood samples from patients treated with chemotherapy and autologous stem cell transplantation (ASCT). Our findings demonstrate that both pre-transplant immune status and post-transplant immune reconstitution are robust predictors of clinical outcomes. Specifically, prior to ASCT, lower counts of regulatory T cells (Tregs) and immature NK cells were associated with a favorable prognosis. Post-transplant, superior outcomes were significantly linked to the reconstitution of γδ T cells and marginal zone B cells, alongside a lower proportion of exhausted T cells. Collectively, these data underscore the critical necessity of integrating routine immune cell profiling with conventional tumor markers to optimize patient management and accurately predict therapeutic responses.

## 1. Introduction

Multiple myeloma (MM) is a hematologic malignancy originating from plasma cells, characterized by the production of monoclonal immunoglobulins and the accumulation of malignant plasma cells within the bone marrow (BM). As the second most common hematologic cancer, MM predominantly affects the elderly, with its global incidence and mortality rates continuing to rise [1]. Current therapeutic regimens for MM encompass conventional chemotherapy and corticosteroids, often combined with immunomodulatory agents or monoclonal antibodies [2]. Despite significant advancements in MM treatment that have achieved clinical complete response (CR) in many patients, the majority will ultimately relapse due to residual myeloma cells. To evaluate the treatment response in MM, traditional criteria established by the International Myeloma Working Group (IMWG) primarily rely on measurements of monoclonal protein (M-protein) in serum and urine, serum-free light chains (sFLC), and BM plasma cell percentage. To enable a more precise assessment of hematologic response, the IMWG incorporated the concept of minimal residual disease (MRD) into its consensus criteria in 2015 [3,4]. MRD assessment has been shown to significantly enhance the evaluation of hematologic responses by providing a quantifiable measure of tumor burden. This allows for timely and effective therapeutic interventions to mitigate the risk of disease progression. Furthermore, prospective randomized clinical trials have established MRD as an independent prognostic factor in MM patients, regardless of their clinical stage or genetic background [5,6,7,8,9].

MM is a genetically complex disease characterized by profound heterogeneity and multifaceted pathogenesis. The immune system plays a pivotal role in MM pathobiology, and is essential for understanding both disease control and progression. While it can mount antitumor responses to suppress cancer growth, it may also paradoxically facilitate tumor development under the influence of malignant cells and an immunosuppressive microenvironment [10]. Immune cells, including lymphocytes, neutrophils, and monocytes, have been reported to be associated with the prognosis of MM patients [11,12]. Studies have demonstrated that analyzing immune cells before and after treatment in MM patients can identify immune phenotypes associated with disease improvement. Furthermore, outcomes after autologous stem cell transplantation (ASCT) have been reported to correlate with specific subsets of these immune cells [13,14,15]. Given the vast number of immune cell subsets and the fine-grained nature of their functions, a comprehensive immune cell profiling in MM patients is beneficial for analyzing disease progression and prognosis.

In this study, we evaluated complete blood count (CBC) parameters associated with prognostic risk in MM patients, with a specific focus on the longitudinal monitoring of immune cell profiles before and after ASCT. We aimed to identify biomarkers indicative of a favorable prognosis. Our results demonstrate that the comprehensive assessment of CBC metrics, MRD status, and immune cell profiling around the time of ASCT holds substantial prognostic value for MM patients. These findings underscore the critical role of regular monitoring in the clinical management of MM, highlighting its potential to optimize therapeutic strategies and improve patient outcomes.

## 2. Materials and Methods

### 2.1. Patients and Study Design

We enrolled 93 patients diagnosed with MM at the first affiliated hospital of Sun Yat-sen University between April 2016 and December 2019. The treatment regimen consisted of bortezomib, adriamycin, and dexamethasone (PAD) induction, followed by ASCT. Post-ASCT consolidation therapy was also administered using the PAD regimen. Subsequently, maintenance therapy consisted of Lenalidomide, whereas high-risk patients received a combination of lenalidomide and bortezomib. CBC was performed for all patients using a BC-6800 Plus fully automated hematology analyzer (Mindray, Shenzhen, China). CBC data were collected at diagnosis, at pre-ASCT (defined as 21–28 days after the last dose of the 4th PAD cycle and within 7 days prior to transplantation), and at 3 months post-ASCT (defined as 90 ± 14 days after stem cell reinfusion). MRD analysis was conducted on BM aspirates obtained at multiple time points: pre-ASCT and every 3 months from 3 to 12 months post-ASCT. All pre-ASCT samples were collected within 7 days prior to transplantation to ensure temporal consistency; post-ASCT follow-up samples were obtained within the planned ±14-day window with no significant temporal deviations. Treatment responses were assessed according to the IMWG criteria, which classify responses as stringent complete response (sCR), complete response (CR), very good partial response (VGPR), partial response (PR), stable disease (SD), and progressive disease (PD). For the purposes of this study, patients achieving sCR, CR, VGPR, PR, or SD were defined as having achieved disease control (DC). Notably, the laboratory personnel and data analysts performing the CBC and MRD assessments were blinded to the patients’ clinical characteristics and treatment outcomes to ensure objectivity.

Based on the results of multi-parameter flow cytometry (MFC), patients were stratified into MRD-positive and MRD-negative groups according to their MRD status at 3 months post-ASCT. Clinical data, including sex, age, Revised International Staging System (RISS) stage, Durie–Salmon (DS) stage, and time to progression (TTP), were collected. TTP and overall survival (OS) were determined via hospital records or telephone follow-up. MRD negativity at 3 months post-ASCT was defined as early MRD negativity.

Additionally, immune cell profiling was performed on fresh peripheral blood (PB) samples collected pre-ASCT and at 3 months post-ASCT from a subset of 33 patients. The 33 patients were selected based on the availability of sufficient cryopreserved peripheral blood mononuclear cells (PBMCs) and were consecutive cases meeting this criterion.

### 2.2. Flow Cytometric Immunophenotyping (FCI) of MRD

BM aspirate samples were collected in EDTA-anticoagulated tubes and processed within 24 h. An optimized 8-color, 2-tube antibody panel comprising cKappa, cLambda, CD81, CD56, CD138, CD19, CD38, and CD45 was utilized to accurately identify phenotypically aberrant, clonal plasma cells. This 2-tube strategy enables MRD detection by specifically confirming light-chain clonality in phenotypically aberrant plasma cells, which are distinguished from normal plasma cells by antigen underexpression (CD19, CD38, CD45, and CD81) or overexpression (CD56 and CD138). Data acquisition was performed on a FACSCanto plus flow cytometer (BD Biosciences, San Jose, CA, USA) and daily instrument quality control was performed using Cytometer Setup and Tracking (CS&T) beads (BD Biosciences) according to the manufacturer’s instructions and EuroFlow standardization protocols [16]. Subsequent data analysis was conducted using Kaluza software v2.3.1 (Beckman Coulter Inc., Brea, CA, USA,). A minimum of 1,000,000 live events were acquired to achieve a potential sensitivity of at least 2 × 10^−5^ (0.002%). The number of viable nucleated cells was systematically recorded, and the limit of detection (LOD) for each sample was calculated using the formula (20/viable nucleated cells) × 100%. Patients were classified as MRD-positive if the percentage of phenotypically aberrant clonal plasma cells was equal to or greater than the sample-specific LOD. Conversely, patients were deemed MRD-negative if phenotypically aberrant clonal plasma cells were either absent or present below the sample-specific LOD. Prior to sample processing, instrument alignment, sensitivity, and spectral compensation were verified using standardized controls, calibrators, procedural controls, and normal peripheral blood samples.

### 2.3. FCI of Immune Cell Profiling

Fresh PB samples were collected prior to ASCT mobilization and at 3 months post-ASCT (median, 101 days) for flow cytometric analysis of immune cell subsets. All fresh peripheral blood (PB) samples were processed and analyzed within 24 h of collection. We utilized the DURAClone IM Immune Function reagent (Beckman Headquarters, Brea, CA, USA), which comprises 50 antibodies distributed across six tubes to identify a total of 66 circulating immune cell subsets. Detailed nomenclature for each subset is provided in Supplementary Table S1.

All samples were analyzed using a 10-color, three-laser Navios flow cytometer (Beckman Coulter), with data files processed via Kaluza software (Beckman Coulter). Daily instrument quality control was rigorously performed on the Navios flow cytometer (Beckman Coulter) prior to sample acquisition. Flow-Check™ fluorospheres were used to verify optical alignment and fluidics stability, while Flow-Set™ fluorospheres were used to standardize photomultiplier tube (PMT) voltages and monitor detector sensitivity. This daily calibration procedure, following the manufacturer’s standardized protocols, ensured consistent instrument performance throughout the entire study, thereby minimizing technical variability and ensuring the reliability of longitudinal data [17].

Absolute immune cell counts were derived by matching the relative proportions obtained from flow cytometry with the corresponding complete blood count (CBC) parameters from the same blood samples. LASSO regression (α = 1) was performed for variable selection. Prior to modeling, all continuous variables were standardized using the Z-score method to ensure that the penalty was applied equally across all features. The optimal regularization parameter (λ) was determined via cross-validation, and the one-standard-error rule (λ.1se) was selected to construct a more parsimonious and robust model.

### 2.4. Statistical Methods

Statistical analyses were performed using SPSS version 25.0 and R version 4.4.2. The normality of data distribution was assessed using the one-sample Kolmogorov–Smirnov test. Normally distributed continuous variables are expressed as *mean* ± standard deviation (*SD*) and were compared between two groups using the independent-samples *t*-test. Non-normally distributed data are presented as median and interquartile range (*IQR*), between-group comparisons were conducted using the Mann–Whitney *U* test, categorical variables are presented as frequencies and percentages, and comparisons among multiple groups were conducted using the chi-square (*χ*^2^) test. Variables independently associated with progression-free survival (PFS) were identified using the Cox proportional hazards regression model. All *p*-values reported in this study were two-sided, with a significance level set at 0.05. CBC parameters were screened using LASSO regression via the glmnet package (version 3.0-2). Variables with non-zero coefficients were selected to construct a prognostic risk score model, which was used to evaluate the predictive value of CBC parameters for MRD status. Statistical analysis of immune cell profiling data was conducted using the DxAI Intelligent Research Platform. Categorical variables were analyzed using the Fisher exact test or chi-square (*χ*^2^) test, while continuous variables were compared using the independent-samples *t*-test or Wilcoxon rank-sum test. A *p*-value < 0.05 was considered statistically significant.

## 3. Results

### 3.1. Patient Characteristics

A total of 93 patients were enrolled in our study, comprising 41 women and 52 men. Table 1 summarizes the demographics and clinical characteristics of the patients at diagnosis, stratified by their MRD status at 3 months post-ASCT. Following ASCT, 37 patients were MRD-positive and 56 were MRD-negative. At initial diagnosis, 23 patients (24.7%) were classified as RISS stage I, 53 patients (57.0%) as stage II, and 17 patients (18.3%) as stage III. Age, sex, β_2_-microglobulin (β_2_MG), hemoglobin (Hb), creatinine (Cr), lactate dehydrogenase (LDH), serum calcium (Ca), and RISS stage were not significantly associated with post-ASCT MRD status ( all *p* > 0.05; Table 1).

### 3.2. Impact of MRD Status on Overall Survival (OS) and PFS

**Figure 1:**
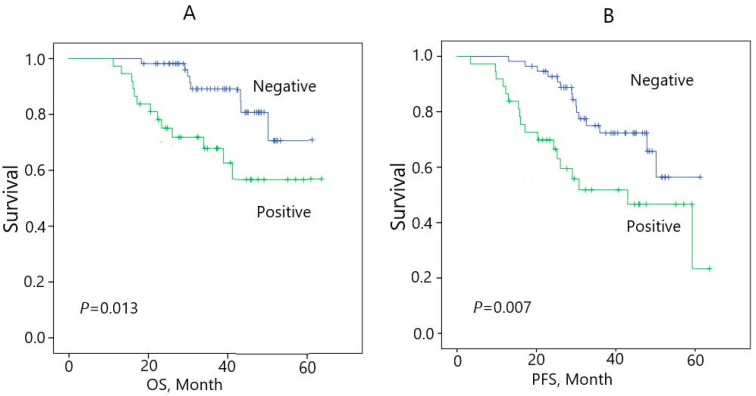
Correlation between post-ASCT MRD status and survival outcomes in patients with multiple myeloma. (**A**) Correlation between post-ASCT MRD status and overall survival (OS). (**B**) Correlation between post-ASCT MRD status and progression-free survival (PFS).

All enrolled MM patients achieved at least a very good partial response (VGPR) following PAD chemotherapy. Both OS and PFS were significantly associated with post-ASCT MRD status (*p* < 0.05; Figure 1). The MRD-positive group had a significantly shorter median OS than the MRD-negative group (32.5 months [range, 21.5–45.2] vs. 39.3 months [range, 29.4–47.6]; *p* = 0.013; Figure 1A). Similarly, the MRD-positive group exhibited a significantly shorter median PFS compared with the MRD-negative group (25.2 months [range, 15.9–43.8] vs. 35.8 months [range, 27.4–47.1]; *p* = 0.007; Figure 1B).

### 3.3. CBC Parameters Closely Associated with MRD Status

**Figure 2:**
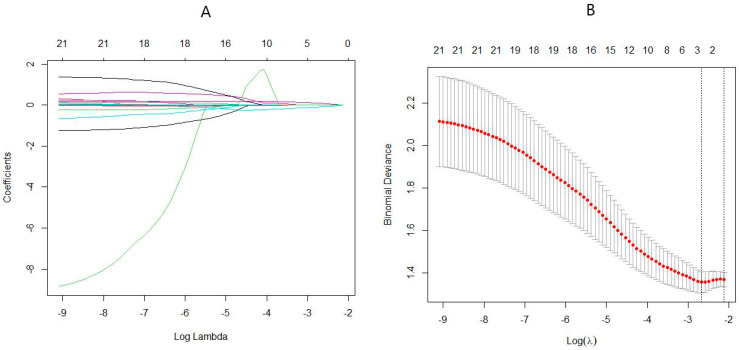
LASSO regression analysis for variable selection. (**A**) Coefficient profiles of the 25 candidate variables. (**B**) Ten-fold cross-validation for tuning parameter selection in the LASSO model. Note: Using LASSO regression and 10-fold cross-validation, three variables (NEU, PLT, and LMR) were identified from the 25 recorded variables as being significantly associated with the risk of MRD positivity.

LASSO regression analysis (Figure 2A) and 10-fold cross-validation (Figure 2B) were performed on 25 variables, including biochemical parameters, CBC parameters, and their derived indices. Three variables were identified as being significantly associated with MRD status at diagnosis: neutrophil count (NEU), platelet count (PLT), and the lymphocyte-to-monocyte ratio (LMR). Specifically, lower NEU or PLT levels, along with a higher LMR, indicated an increased risk of MRD positivity.

### 3.4. Construction and Evaluation of the CBC-Based Prognostic Model for Patients with Multiple Myeloma

**Figure 3:**
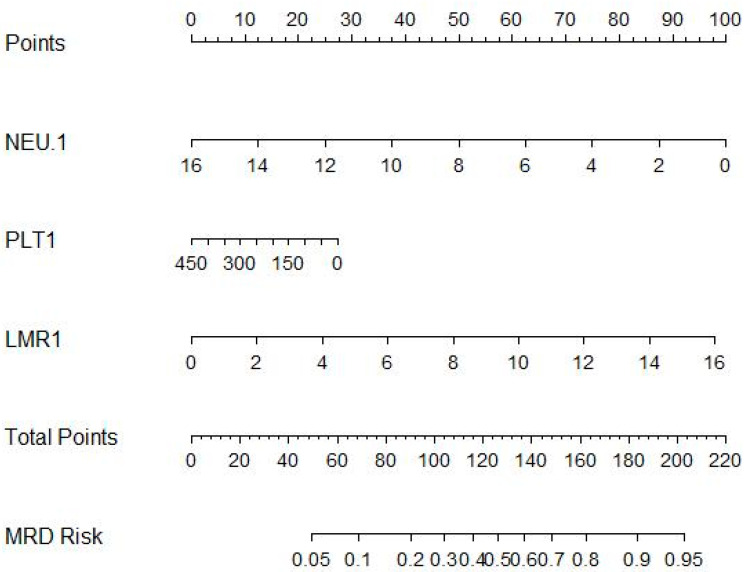
Nomogram for predicting the risk of MRD positivity in patients with multiple myeloma. Note: Lower NEU or PLT levels, along with a higher LMR, correspond to higher total points, indicating an increased risk of MRD positivity.

**Figure 4:**
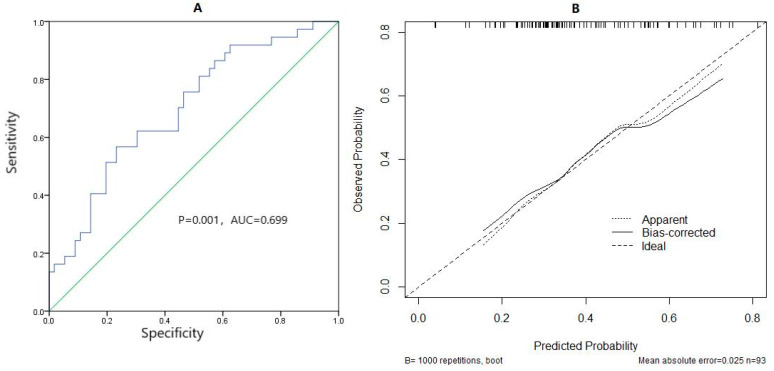
Performance evaluation of the prognostic model. (**A**) Receiver operating characteristic (ROC) curve demonstrating the predictive accuracy of the model. (**B**) Calibration curve assessing the agreement between predicted and observed probabilities. Note: ROC curve demonstrated excellent predictive performance, with an area under the curve (AUC) of 0.699 (*p* = 0.001). The model exhibited good discriminative ability, yielding a C-index of 0.723. Calibration evaluation revealed that the predicted probabilities were in good agreement with the ideal reference line.

Based on the variables screened by LASSO regression, a predictive model was constructed to estimate the risk of MRD positivity in MM patients (Figure 3). Using the diagnostic values of NEU, PLT, and LMR, the probability of early MRD positivity post-ASCT can be visually calculated via a nomogram. The receiver operating characteristic (ROC) curve demonstrated excellent predictive performance, with an area under the curve (*AUC*) of 0.699 (*p* = 0.001; Figure 4A). Furthermore, the model exhibited good discriminative ability, yielding a C-index of 0.723. Calibration evaluation revealed that the predicted probabilities were in good agreement with the ideal reference line (Figure 4B).

### 3.5. Immune Cell Profiles Prior to ASCT

Prior to ASCT, the counts of CD4/CD8 double-negative T cells (DNTs), regulatory T cells (Tregs), and CD16^+^ CD56^high^ natural killer (NK) cells were significantly lower in MRD-negative patients compared to MRD-positive patients (all *p* < 0.05). Specifically, the counts were (0.015 ± 0.014) × 10^9^/L vs. (0.032 ± 0.012) × 10^9^/L for DNTs, and (0.012 ± 0.010) × 10^9^/L vs. (0.040 ± 0.019) × 10^9^/L for Tregs. Additionally, the percentages of CD16^+^ CD56^high^ NK cells were 0.240% (range: 0.122–0.502%) vs. 1.970% (range: 0.660–3.790%), respectively (Table 2).

Prior to ASCT, disease progression in MM patients was significantly associated with elevated counts of PD1^+^ memory CD4^+^ T cells (T4CM) and CD8^+^ HLA-DR^+^ T cells. Specifically, the progressive group exhibited significantly higher levels of PD1^+^T4CM and CD8^+^ HLA-DR^+^ T cells compared to the non-progressive group [PD1^+^T4CM: (48.966 ± 22.905)% vs. (28.126 ± 12.354)%; CD8^+^ HLA-DR^+^ T cells: (84.016 ± 11.822)% vs. (68.392 ± 13.164)%; both *p* < 0.05] (Table 2). Conversely, the counts of naive CD8^+^ T cells and naive CD8^+^ CD28^+^ CD27^+^ T cells were significantly lower in the progressive group than in the non-progressive group [naive CD8^+^ T cells: 4.640% (range: 2.390–5.750%) vs. 12.070% (range: 7.000–23.200%); naive CD8^+^ CD28^+^ CD27^+^ T cells: (88.514 ± 8.544)% vs. (95.944 ± 2.787)%; both *p* < 0.05] (Table 2). Comprehensive data on immune cell profiles prior to ASCT are summarized in Table 2 and Supplementary Table S2.

### 3.6. Immune Cell Profiles Post-ASCT

Post-ASCT, elevated levels of activated and exhausted T lymphocytes were significantly associated with disease progression. Specifically, the proportion of HLA-DR^+^ CD4^+^ T cells was significantly higher in the progressive group compared to the non-progressive group [(76.610 ± 17.384) % vs. (59.667 ± 10.372) %; *p* < 0.05] (Table 3). Conversely, the percentage of CD4^+^ central memory T cells (T4CM) at 3 months post-ASCT was significantly lower in the progressive group [(38.204 ± 14.839) % vs. (53.315 ± 8.565) %; *p* < 0.05] (Table 3). Furthermore, the ratio of (naive T cells + TCMs) to (TEMs + effector T cells) was significantly higher in the non-progressive group compared to the progressive group (0.489 ± 0.085 vs. 0.338 ± 0.176; *p* < 0.05) (Table 3).

Furthermore, MRD-negative patients exhibited significantly higher levels of γδ T cells than MRD-positive patients [0.052% (range: 0.043–0.117%) vs. 0.019% (range: 0.007–0.032%); *p* < 0.05] (Table 3). Conversely, the proportion of marginal zone B cells was significantly lower in the progressive group compared to the non-progressive group [(1.586 ± 1.428) vs. (2.800 ± 0.760) %; *p* < 0.05] (Table 3). Comprehensive data on immune cell profiles post-ASCT are summarized in Table 3 and Supplementary Table S3.

## 4. Discussion

MRD plays a pivotal role in the clinical management of MM and is unequivocally established as a robust prognostic biomarker [18,19]. For instance, Gupta et al. reported a median PFS of 24 months in MRD-positive patients, whereas the median PFS was not yet reached in the MRD-negative cohort (*p* < 0.05) [20]. Consistent with these findings, our study demonstrated that patients who achieved early MRD negativity exhibited significantly prolonged PFS compared to their MRD-positive counterparts (36 months vs. 25 months, *p* < 0.05). Collectively, these data underscore the profound prognostic value of early MRD assessment post-ASCT. Consequently, MRD evaluation has been increasingly advocated to enhance the sensitivity of response evaluation and is now proposed as a reliable surrogate endpoint for PFS in MM. While MRD 3 months post-ASCT is a strong surrogate [Appendix A and Appendix B], sustained MRD negativity (conversion from positive to negative or maintaining negativity) carries different prognostic weight compared to transient negativity.

Immune homeostasis serves as a critical determinant in the equilibrium between MM dormancy and progression, driven by dynamic shifts in the surrounding immune microenvironment. Deciphering these complex tumor–immune interactions necessitates an evaluation of the broader systemic immune context, extending beyond the immediate tumor microenvironment. Therefore, our study longitudinally tracked immune cell profiles in MM patients pre- and post-ASCT, thereby elucidating the reconstitution of the immune landscape and its prognostic implications for disease progression.

Previous studies have established that the absolute peripheral blood lymphocyte count, neutrophil-to-lymphocyte ratio (NLR), lymphocyte-to-monocyte ratio (LMR), and platelet-to-lymphocyte ratio serve as valuable prognostic biomarkers in MM [21,22,23,24]. Our study demonstrated that lower neutrophil (NEU) and platelet (PLT) counts, coupled with an elevated LMR at diagnosis, were associated with an increased risk of MRD positivity. Indeed, reduced NEU and PLT counts prior to treatment initiation are recognized as adverse prognostic factors in MM. Similarly, lymphopenia is widely considered a poor prognostic indicator across various malignancies [11,21]. In our research, post-ASCT lymphocyte levels were significantly lower, whereas NEU counts were higher in the MRD-positive group compared to the MRD-negative group. Consequently, the MRD-positive cohort exhibited a higher post-ASCT NLR, suggesting that an elevated NLR predicts unfavorable clinical outcomes, which aligns with the existing literature [21,22,24]. Furthermore, while an elevated LMR has been previously associated with a favorable prognosis in MM [21,22], our findings revealed a paradox: a higher LMR at diagnosis was linked to an increased risk of MRD positivity. Notably, this ratio decreased in the MRD-positive group post-ASCT. We hypothesize that adequate baseline monocyte levels are required prior to ASCT to exert their intrinsic phagocytic and cytotoxic functions; the subsequent depletion or dysregulation of these cells may ultimately contribute to a poorer prognosis.

As key components of the innate immune system, natural killer (NK) cells are essential for immune surveillance and the elimination of infected or malignant cells. Furthermore, they actively modulate the functions of T cells, macrophages, and dendritic cells. Beyond the predominant cytotoxic NK cell subset (CD16^+^ CD56^+^, comprising approximately 90% of total NK cells), there exists a minor population of cytokine-secreting or immature NK cells (CD16^+^ CD56^high^, ~ 10%). These cells produce diverse cytokines and chemokines that can either restrain or amplify immune responses, recruit leukocytes to sites of inflammation, and shape adaptive immunity. Previous studies have demonstrated that peripheral blood NK cell counts decline as myeloma advances, whereas the proportion of immature NK cells increases in patients with relapsed/refractory MM [25,26]. Consistent with these findings, our study revealed that pre-ASCT NK cell counts were significantly lower in the MRD-positive cohort compared to the MRD-negative cohort. Moreover, elevated levels of immature NK cells both before and after ASCT were indicative of an unfavorable prognosis in MM patients.

Dendritic cells (DCs) are professional antigen-presenting cells that play an important role in the immunotherapy of MM. Notably, the proteasome inhibitor bortezomib induces immunogenic cell death (ICD), which facilitates the activation of DCs via enhanced exposure to tumor antigens. However, DC deficiencies have been frequently reported in patients with MM [27]. In our study, disease progression was significantly associated with elevated levels of plasmacytoid DCs (pDCs) prior to ASCT. The distinct prognostic implications of different DC subsets in MM warrant further investigation.

γδ T cells, a unique subset of innate-like T lymphocytes, typically comprise 1–10% of the total peripheral T cell pool. In our cohort, MRD-negative patients exhibited a significant expansion of γδ T cells at three months post-ASCT, which aligns with the current literature. High frequencies of γδT cells have been consistently associated with prolonged disease-free survival in both pediatric and adult oncology patients [28]. Mechanistically, γδ T cells undergo rapid early reconstitution within two months post-ASCT. The majority of these early-recovered cells originate from donor-derived γδ1 and γδ2 subsets and predominantly exhibit a CD27^+^/CD45RA^−^ central memory phenotype. This distinct phenotypic profile is crucial for conferring early protective immunity against viral and bacterial pathogens, as well as residual malignant cells.

Beyond γδ T cells, CD4^+^ T cell subsets also play a pivotal role in mediating anti-tumor immunity. For instance, CD4+ central memory T cells (T4CM) are critical for lymph node homing, DC activation, and subsequent differentiation into CD4^+^ effector T cells. Consistent with this, our study demonstrates that higher post-ASCT T4CM frequencies serve as a favorable prognostic indicator in MM. Conversely, analyses prior to ASCT revealed that specific T4CM subsets, namely CD28^−^CD27^−^ and programmed death-1 (PD-1)^+^T4CM cells, were significantly enriched in patients with MRD positivity and disease progression. Furthermore, the proportion of regulatory T cells (Tregs) was also elevated in the MRD-positive cohort prior to ASCT. Tregs are primarily responsible for maintaining immune homeostasis through the suppression of immune responses and the induction of immune tolerance [29].

As an inhibitory receptor, the upregulation of PD-1 expression is closely associated with T cell exhaustion. This exhaustion impairs normal T cell differentiation and abrogates cytotoxic effector functions, thereby contributing to a poor prognosis. Furthermore, treatment-induced immune dynamics, such as those following ASCT, have been shown to drive T cell heterogeneity in MM, promoting the emergence of exhausted or senescent T cell phenotypes [30]. Within the CD8^+^ effector memory T cell (T8EM) compartment, only the CD28^−^CD27^−^ subset was significantly elevated in the post-ASCT MRD-positive group, whereas the other subsets (CD28^−^CD27^+^ and CD28^+^ CD27^+^ T8EM) were diminished. Notably, the T8EM population also encompasses exhaustion- and senescence-associated subsets (e.g., PD1^+^ and CD28^−^CD27^+^ T8EM), which aligns with the post-transplant MRD status and suggests that therapeutic interventions profoundly reshape T cell heterogeneity. Sustained anti-tumor immunity relies on the long-term survival of memory CD8^+^ T cells; this may explain the higher frequencies of CD8^+^ central memory T cells (T8CM) observed in the post-ASCT MRD-negative cohort. The relative contraction of naive and central memory T cell pools, coupled with the terminal differentiation toward effector memory and effector T cells, is indicative of T cell exhaustion. To quantify this exhausted state, we calculated the ratio of (naive T cells + central memory T cells) to (effector memory T cells + effector T cells). A reduced post-ASCT ratio in patients with progressive disease suggests that profound T cell exhaustion is intricately linked to an unfavorable prognosis [31,32].

Although our study did not find a significant association between disease progression and the total CD19^+^ B cell count, subset analysis revealed that patients with a poor prognosis exhibited significantly lower proportions of marginal zone (MZ) B cells post-ASCT. Recent studies have demonstrated that MZ B cells can engulf dendritic cells (DCs) via a process known as pulverization, thereby acquiring MHC class II molecules bound to complement C3 and presenting them as antigens to CD4^+^ T cells [33]. Furthermore, given that MM is fundamentally a clonal malignancy of plasma cells derived from the B-cell lineage, the precise mechanisms by which MZ B cells influence MM prognosis warrant further investigation.

A central challenge in post-ASCT management is steering the reconstituting immune system towards an anti-tumor direction. Our identification of specific immune phenotypes associated with favorable outcomes provides theoretical targets for active immunomodulation. For instance, strategies aimed at enhancing the expansion and persistence of [central memory T cells, γδ T cells and marginal zone B cells] while mitigating the accumulation of immunosuppressive populations [Tregs, immature NK cells] could be explored. Future therapeutic implementations might include post-transplant adoptive cell therapy, targeted cytokine administration, or the strategic timing of immunomodulatory drugs (IMiDs) to coincide with critical windows of immune plasticity, thereby actively shaping a protective anti-myeloma microenvironment.

It is important to acknowledge that post-ASCT consolidation and maintenance therapies significantly influence long-term prognosis and immune reconstitution kinetics. In our study, the majority of patients received Lenalidomide maintenance, which is known to exert immunomodulatory effects, including the enhancement of T-cell and NK-cell function and the reduction in Tregs. While this could theoretically confound the analysis of spontaneous immune recovery, our study design aimed to capture the immune landscape under standard-of-care conditions, which reflects real-world clinical practice. Furthermore, although maintenance therapy modulates the immune microenvironment, previous studies suggest that the depth and quality of immune reconstitution early post-transplant (e.g., at day +100) remain independent prognostic factors regardless of subsequent maintenance.

The design of this pilot study was carefully structured to balance feasibility with scientific rigor. The inclusion of 93 patients for longitudinal sampling over a 3.5-year period was driven by the logistical challenges of obtaining serial bone marrow samples in MM patients, yet it provides a robust foundation for observing temporal immune dynamics. The decision to perform in-depth immune cell profiling on a subset of 33 patients was based on resource constraints and the exploratory nature of this phase; however, this cohort was statistically powered to detect significant differences in key immune subsets. The 3 months post-transplantation timepoint was selected as it represents a critical biological window of early immune reconstitution, preceding the potential onset of relapse. Furthermore, our statistical models were intentionally designed to prioritize hypothesis generation over confirmatory validation, acknowledging the exploratory scope of this pilot phase.

Our study has several limitations. First, due to resource constraints, the cohort size for the immune cell profiling analysis was limited. Second, we did not evaluate the prognostic impact of dynamic changes in immune markers pre- and post-transplantation. Additionally, it should be noted that the 3-month timepoint reflects a specific window of immune reconstitution rather than a fully established steady state. Third, our analysis of immune cell subsets was hypothesis-generating; therefore, corrections for multiple testing were not applied. These findings should be interpreted with caution and validated in future independent cohorts. Fourth, we did not comprehensively evaluate the interplay between lenalidomide therapy and immune reconstitution, nor their specific impacts on patient outcomes. Finally, the performance metrics derived from our LASSO regression should be interpreted as apparent values. Due to the exploratory nature of this study, we did not conduct internal validation using Bootstrap resampling (1000 iterations) to obtain a bias-corrected C-index.

To confirm these pilot findings, future studies should employ a prospective, multi-center longitudinal design. An optimal confirmatory study would require a larger, uniformly treated cohort (n > 300) with standardized sample collection at predefined milestones (e.g., pre-transplant, day +30, +100, +6 months, and +12 months). Crucially, future protocols should integrate comprehensive immune profiling with detailed longitudinal treatment records to account for confounding variables, such as maintenance therapy (e.g., lenalidomide) and relapse interventions. Incorporating advanced statistical methods, including time-dependent covariates and machine learning algorithms, will be essential to map the complex interplay between dynamic immune reconstitution and long-term survival outcomes.

## 5. Conclusions

In conclusion, the assessment of CBC, MRD status, and immune cell profiles both prior to and following ASCT holds significant prognostic value for patients with MM. In conclusion, this pilot study identifies distinct trajectories of immune reconstitution post-ASCT that are intimately linked to PFS in MM patients. Specifically, the early recovery of [central memory T cells, γδ T cells and marginal zone B cells] and the delayed restoration of [Tregs, immature NK cells] serve as critical functional biomarkers for disease control. These findings underscore the necessity of moving beyond standard clinical parameters to incorporate dynamic immune monitoring. Clinically, these immune signatures provide a rationale for active implementation in treatment strategies, such as personalized maintenance therapy or targeted immunomodulation aimed at steering the immune balance in an anti-tumor direction. Future prospective, multi-center studies are warranted to validate these immune biomarkers and translate them into actionable clinical protocols.

## Figures and Tables

**Table 1:** Patient demographics and clinical characteristics.

Characteristic	MRD-Positive (n = 37)	MRD-Negative (n = 56)	*p*
Age, median (IQR), years	53 (49–59)	54 (46–60)	0.734
Male sex, n (%)	24 (64.9)	28 (50.0)	0.158
R-ISS stage, n (%)			0.874
I	8 (21.6)	15 (26.8)	
II	24 (64.9)	29 (51.8)	
III	5 (13.5)	12 (21.4)	
Hb, median (IQR), g/L	102.0 (80.5–115.0)	97.5 (80.8–114.5)	0.371
β_2_MG, median (IQR), mg/L	3712.8 (2392.3–6114.1)	4043.3 (2272.8–8150.4)	0.672
Cr, median (IQR), μmol/L	84.0 (64.5–129.5)	81.0 (65.0–165.5)	0.721
LDH, median (IQR), U/L	186.0 (155.5–230.5)	166.5 (121.5–215.0)	0.110
Ca, median (IQR), mmol/L	2.3 (2.1–2.5)	2.3 (2.2–2.4)	0.680

Note: Mann–Whitney *U* test for continuous variables; chi-square (*χ*^2^) test for categorical variables; R-ISS, Revised International Staging System; IQR, interquartile range.

**Table 2:** Key immune cell subsets predicting MRD status and prognosis prior to ASCT in patients with multiple myeloma.

Immune Cell Subsets Prior to ASCT	Prognosis		*p*	MRD Status Prior to ASCT		*p*
	Non-Progressive (n = 28)	Progressive (n = 5)		Negative (n = 9)	Positive (n = 24)	
DNTs	0.025 ± 0.016	0.025 ± 0.015	0.99	0.015 ± 0.014	0.032 ± 0.012	<0.01 **
NK cells	15.262 ± 5.949	13.814 ± 5.953	0.59	19.093 ± 5.826	12.013 ± 4.054	<0.01 **
CD16^+^ CD56^high^ NK cells	0.660 (0.260–1.970)	1.795 (0.287–4.645)	0.37	0.240 (0.122–0.502)	1.970 (0.660–3.790)	<0.01 **
PD1^+^T4CM cells	28.126 ± 12.354	48.966 ± 22.905	0.04 *	29.893 ± 9.672	42.482 ± 28.314	0.33
CD8^+^ CD57^+^ T cells	0.122 ± 0.079	0.138 ± 0.066	0.63	0.079 ± 0.060	0.158 ± 0.065	0.01 *
Naive CD8^+^ T cells	12.070 (7.000–23.200)	4.640 (2.390–5.750)	0.03 *	16.940 (8.090–26.070)	5.865 (4.768–6.835)	<0.01 **
PD1^+^T8CM cells	0.013 ± 0.010	0.016 ± 0.006	0.44	0.008 ± 0.006	0.018 ± 0.008	<0.01 **
Naive CD8^+^CD28^+^CD27^+^ T cells	95.944 ± 2.787	88.514 ± 8.544	0.03 *	96.200 ± 1.185	93.120 ± 2.608	<0.01 **
T8CM CD28^+^ CD27^−^ cells	4.983 ± 2.894	2.712 ± 1.104	0.05 *	4.660 (3.027–8.227)	3.300 (2.590–3.730)	0.12
CD8^+^ HLA-DR^+^T cells	68.392 ± 13.164	84.016 ± 11.822	0.05 *	71.508 ± 13.292	72.260 ± 18.908	0.94
T8E CD28^−^ CD27^−^ cells	0.089 ± 0.075	0.094 ± 0.054	0.85	0.044 (0.019–0.061)	0.104 (0.063–0.177)	0.02 *
Tregs	0.029 ± 0.019	0.029 ± 0.026	0.96	0.012 ± 0.010	0.040 ± 0.019	<0.01 **
Memory Tregs	0.025 ± 0.018	0.027 ± 0.024	0.83	0.009 ± 0.009	0.037 ± 0.017	<0.01 **
pDCs	0.070 (0.050–0.080)	0.120 (0.110–0.155)	0.01 *	0.090 (0.062–0.150)	0.090 (0.080–0.120)	0.91

Note: Data are presented as mean ± standard deviation or median (interquartile range, IQR). All variables are expressed as percentages (%) unless otherwise indicated; absolute counts are expressed as ×10^9^/L. * *p* < 0.05, ** *p* < 0.01. ASCT, autologous stem cell transplantation; MRD, minimal residual disease; DNTs, CD4/CD8 double-negative T cells; pDCs, plasmacytoid dendritic cells; T4CM, CD4^+^ central memory T cells; T8CM, CD8^+^ central memory T cells; T8E, CD8^+^ effector T cells.

**Table 3:** Key immune cell subsets predicting MRD status and prognosis post-ASCT in patients with multiple myeloma.

Immune Cell Subsets Post-ASCT	Prognosis		*p*	MRD Status Post-ASCT		*p*
	Non-Progressive (n = 28)	Progressive (n = 5)		Negative (n = 24)	Positive (n = 9)	
Granulocytes	46.776 ± 10.376	55.924 ± 22.530	0.29	44.381 ± 10.883	63.998 ± 19.213	0.03 *
Lymphocytes	40.150 ± 12.252	31.456 ± 19.365	0.30	42.426 ± 11.987	22.210 ± 13.737	0.02 *
transitional B cell	16.106 ± 8.103	28.606 ± 6.481	0.01 *	19.594 ± 10.284	24.367 ± 7.397	0.42
marginal zone B cell	2.800 ± 0.760	1.586 ± 1.428	0.05 *	2.661 ± 0.930	1.390 ± 1.137	0.05 *
CD16^+^ CD56high NK cells	3.855 ± 4.755	3.786 ± 3.762	0.98	0.945 (0.665–1.805)	7.285 (3.865–10.928)	0.02 *
CD3^+^ T cells	1.533 ± 1.261	0.917 ± 0.701	0.33	1.225 (1.030–1.913)	0.744 (0.471–0.882)	0.01 *
CD4^+^ HLA-DR^+^ T cells	59.667 ± 10.372	76.610 ± 17.384	0.03 *	65.642 ± 15.168	67.875 ± 16.867	0.81
T4CM	53.315 ± 8.565	38.204 ± 14.839	0.03 *	47.048 ± 11.512	47.657 ± 17.072	0.94
T4CM CD28^+^ CD27^−^ cells	0.016 ± 0.008	0.006 ± 0.003	0.02 *	0.014 ± 0.009	0.008 ± 0.006	0.21
T8 CD28^+^ CD27^+^ cells	0.493 ± 0.847	0.181 ± 0.194	0.44	0.280 (0.189–0.419)	0.100 (0.075–0.113)	0.02 *
T8EM CD28^−^CD27^−^ cells	35.709 ± 13.534	44.704 ± 11.293	0.23	34.705 ± 12.886	50.180 ± 8.426	0.05 *
CD3^+^ TCR γδ^+^ T cells	0.073 ± 0.076	0.065 ± 0.071	0.85	0.052 (0.043–0.117)	0.019 (0.007–0.032)	0.04 *
TN+TCM/TEM+TE ratio	0.489 ± 0.085	0.338 ± 0.176	0.04 *	0.441 ± 0.169	0.416 ± 0.119	0.75
CD8^+^PD1^+^/CD4^+^PD1^+^ ratio	4.144 ± 2.342	2.636 ± 2.129	0.25	4.197 (3.438–5.245)	1.700 (1.264–2.082)	<0.01 **

Note: Data are presented as mean ± standard deviation or median (interquartile range, IQR). All variables are expressed as percentages (%) unless otherwise indicated; absolute counts are expressed as ×10^9^/L. * *p* < 0.05, ** *p* < 0.01. ASCT, autologous stem cell transplantation; MRD, minimal residual disease; T4CM, CD4^+^ central memory T cells; T8EM, CD8^+^ effector memory T cells; TN, naive T cells; TCM, central memory T cells; TEM, effector memory T cells; TE, effector T cells.

**Figure A1:**
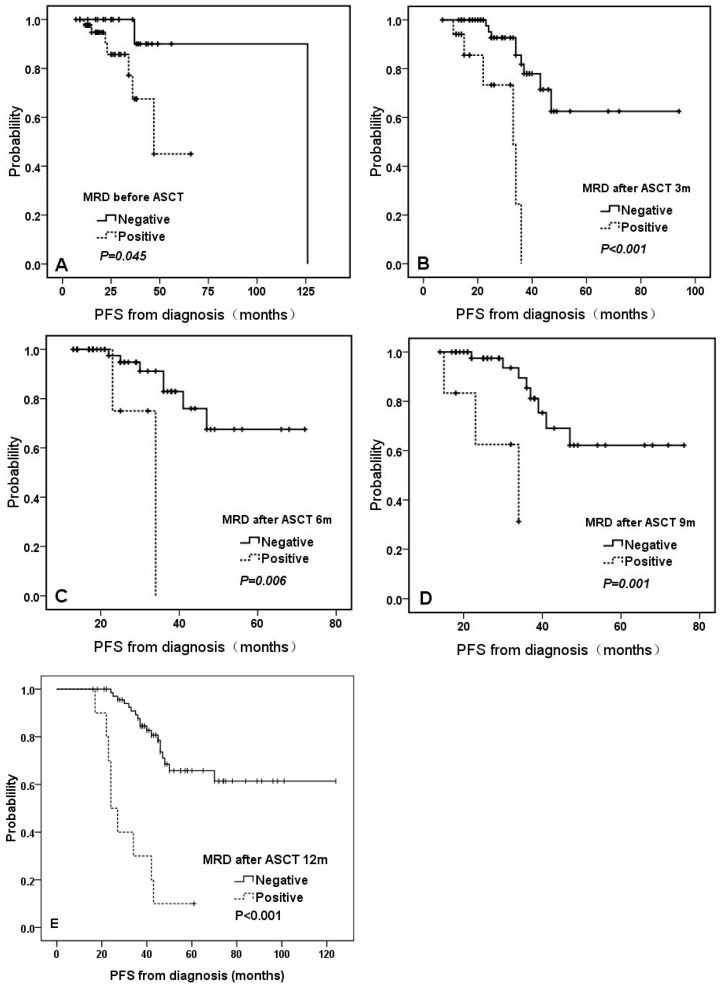
Progression-free survival analysis with different MRD status before ASCT (**A**), after ASCT 3M (**B**), after ASCT 6M (**C**), after ASCT 9M (**D**) and after ASCT 12M (**E**). MRD-negative patients had longer PFS than MRD-positive patients in all group.

**Figure A2:**
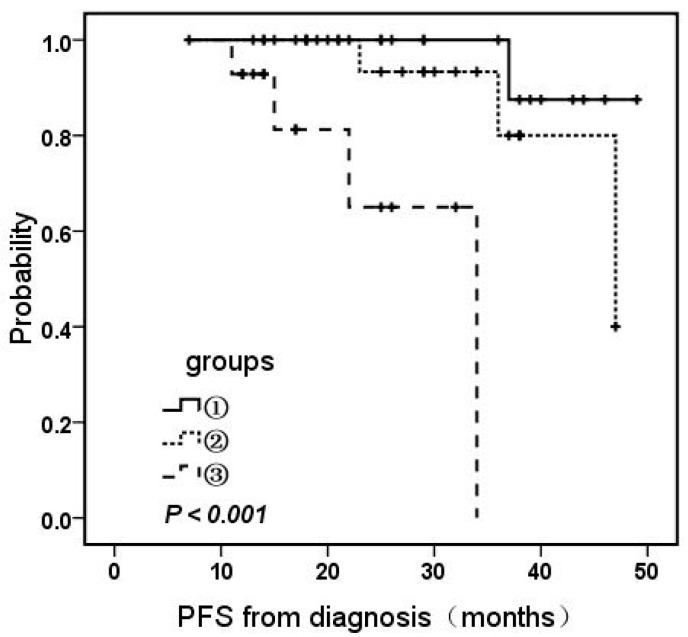
Progression-free survival analysis with different MRD status before and after ASCT.① MRD-negative both before ASCT and after ASCT 3M, ② MRD-positive before ASCT and negative after ASCT 3M, ③ MRD-positive both before ASCT and after ASCT 3M. MRD-negative patients both before ASCT and after ASCT 3M. Group ① had the best PFS.

## Data Availability

The data presented in this study are available upon request from the corresponding author due to privacy restrictions.

## Supplementary Materials

The following supporting information can be downloaded at: https://www.mdpi.com/article/10.3390/cancers18152389/s1. Table S1: Immune cell profiling panels; Table S2: Immune cell profiles and the relationship with disease progression and MRD status prior to ASCT; Table S3: Immune cell profiles and the relationship with disease progression and MRD status post-ASCT.

## Abbreviations

The following abbreviations are used in this manuscript:

MM	Multiple myeloma
MRD	Minimal residual disease
CBC	Complete blood count
PAD	Bortezomib/adriamycin/dexamethasone
ASCT	Autologous stem cell transplantation
NEU	Neutrophil count
PLT	Platelet count
LMR	Lymphocyte monocyte ratio
PFS	Progression-free survival
OS	Overall survival
ROC	Receiver operating characteristic
AUC	Area under the curve
FCI	Flow cytometric immunophenotyping

## Appendix A

### Appendix A.1

**Table A1:** Impact of MRD status at different time points on PFS.

MRD Status at Different Times	Patient demographics		PFS	
	Cases	Relapse	Median, M	*p*
**Before ASCT**	68	9		0.04
Negative	22	2	126	
Positive	46	7	47	
**After ASCT 3M**	75	15		<**0.01**
Negative	58	9	NR	
Positive	17	6	33	
**After ASCT 6M**	64	9		<**0.01**
Negative	56	7	NR	
Positive	8	2	34	
**After ASCT 9M**	54	11		<**0.01**
Negative	48	8	NR	
Positive	6	3	34	
**After ASCT 12M**	81	28		<**0.01**
Negative	71	19	NR	
Positive	10	9	32	

Note: M, months; NR, not reach; ***p***, MRD-negative versus positive rate.

### Appendix A.2

**Table A2:** Impact of MRD status before and after ASCT on PFS.

Group	MRD Before ASCT	MRD After ASCT 3M	Cases	Relapse	PFS
①	Negative	Negative	17	1	NR
②	Positive	Negative	26	3	47
③	Positive	Positive	14	4	34

Note: NR, not reach.

### Appendix A.3

**Table A3:** Multivariate analysis of independent prognostic factors on PFS using Cox’s proportional hazard model.

	B	SE	Wald	Df	Sig.	Exp(B)	95.0% CI for Exp(B)	
							Lower	Upper
MRD after ASCT 3M	−5.152	1.678	9.427	1	0.002	0.006	0.000	0.155
subtype	−0.921	0.423	4.731	1	0.030	0.398	0.174	0.913
Response before ASCT	−1.641	0.777	4.455	1	0.035	0.194	0.042	0.889
ISS	−0.268	0.877	0.093	1	0.760	0.765	0.137	4.267
MRD before ASCT	2.008	1.315	2.331	1	0.127	7.447	0.566	98.010
