# Supplementary material for: The Prognostic Value of Combined Complete Blood Count and Immune Cell Profiling in Patients with Multiple Myeloma Treated with Chemotherapy Sequential Transplantation
Source: Cancers (Basel). 2026 Jul 24;18(15):2389. doi: 10.3390/cancers18152389 (PMC13465182; doi:10.3390/cancers18152389)
Supplement: Supplementary file 1 [file cancers-18-02389-s001.zip › cancers-4420671-supplementary.pdf]

The prognostic value of combined CBC and immune cell profiling in patients with multiple myeloma treated with PAD sequential transplantation

Jiang Zhang[#], Yao Chen[#], Yaoming Chen, Mengzhi Hong, Peisong Chen, Junxun Li[*], Juan Ouyang [*]

Department of Laboratory Medicine, The First Affiliated Hospital of Sun Yat-sen University, Guangzhou, China

# These authors contributed equally to this work.

*Corresponding author. *E-mail addresses:* lijunx5@mail.sysu.edu.cn (J. Li), ouyangj2@mail.sysu.edu.cn (J. Ouyang).

Table S1. Immune cell profiling panels

Immune cell phenotype	Epitopes tested	Denominator
Basic		
Leucocytes	$CD45^+$	-
Granulocytes	$CD45^+$ SSC^{high}	Leucocytes
T cells	$CD3^+$	Lymphocytes
B cells	$CD19^+$ $CD3^-$	Lymphocytes
All monocytes	$CD14^+$	Leucocytes
Classical monocytes	$CD14^{high}$ $CD16^-$	Monocytes
Intermediate monocytes	$CD14^{high}$ $CD16^+$	Monocytes
NK-cells	$CD3^-$ $CD56^+$	Lymphocytes
Mature NK-cells	$CD3^-$ $CD56^{dim}$	NK-cells
Immature NK-cells	$CD3^-$ $CD56^{high}$	NK-cells
B cells		
Naive B cells	$CD19^+$ $CD27^-$ IgD^+	B cells
Transitional B cells	$CD19^+$ $CD27^-$ $CD38^{high}$ IgM^+ $CD24^+$	B cells
Marginal zone B	$CD19^+$ $CD27^+$ IgD^+	B cells
Memory B cells	$CD19^+$ $CD27^+$ $CD38^{dim}$	B cells
T cells		
$CD4^+$ cells	$CD3^+$ $CD4^+$	T cells
Naive $CD4^+$ T cells	$CD3^+$ $CD4^+$ $CCR7^+$ $CD45RA^+$	$CD4^+$ cells
Central memory $CD4^+$ T cells ($CD4^+$TCM)	$CD3^+$ $CD4^+$ $CCR7^+$ $CD45RA^-$	$CD4^+$ cells
Effector memory $CD4^+$ T cells ($CD4^+$TEM)	$CD3^+$ $CD4^+$ $CCR7^-$ $CD45RA^-$	$CD4^+$ cells
Differentiated/senescent $CD4^+$ T cells	$CD3^+$ $CD4^+$ $CD27^-$ / $CD3^+$ $CD4^+$ $CD28^-$ / $CD3^+$ $CD4^+$ $CD57^+$	$CD4^+$ cells
Exhausted $CD4^+$ T cells	$CD3^+$ $CD4^+$ $PD1^+$	$CD4^+$ cells
$CD8^+$ cells	$CD3^+$ $CD8^+$	T cells
Naive $CD8^+$ T cells	$CD3^+$ $CD8^+$ $CCR7^+$ $CD45RA^+$	$CD8^+$ cells
Central memory $CD8^+$ T cells ($CD8^+$TCM)	$CD3^+$ $CD8^+$ $CCR7^+$ $CD45RA^-$	$CD8^+$ cells
Effector memory $CD8^+$ T cells ($CD8^+$TEM)	$CD3^+$ $CD8^+$ $CCR7^-$ $CD45RA^-$	$CD8^+$ cells

Differentiated/senescent $CD8^+$ T cells	$CD3^+$ $CD8^+$ $CD27^-$ / $CD3^+$ $CD8^+$ $CD28^-$ / $CD3^+$ $CD8^+$ $CD57^+$	$CD8^+$ cells
Exhausted $CD8^+$ T cells	$CD3^+$ $CD8^+$ $PD1^+$	$CD8^+$ cells
$CD4^+$:$CD8^+$ ratio	$CD3^+$ $CD4^+$: $CD3^+$ $CD8^+$	-
Double-positive T cells/DPT	$CD3^+$ $CD4^+$ $CD8^+$	$CD3^+$ cells
Double-negative T cells/DNT	$CD3^+$ $CD4^-$ $CD8^-$	$CD3^+$ cells
TCR		
TCR-αβ $^+$T cells	TCR-$\alpha\beta^+$ $CD3^+$	T cells
TCR-αβ $^+CD4^+$ T cells	TCR-$\alpha\beta^+$ $CD3^+$ $CD4^+$	TCR-αβ $^+$T cells
TCR-αβ $^+CD8^+$ T cells	TCR-αβ $^+CD3^+$ $CD8^+$	TCR-$\alpha\beta^+$ T cells
TCR-$\gamma\delta^+$ T cells	TCR-γδ $^+CD3^+$	T cells
TCR-γδ $^+CD4^+$ T cells	TCR-γδ $^+CD3^+$ $CD4^+$	TCR-γδ $^+$T cells
TCR-γδ $^+CD8^+$ T cells	TCR-γδ +CD3+ CD8+	TCR-γδ $^+$T cells
DCs		
Dendritic cells	Lin^- $HLA\text{-}DR^+$	Leucocytes
Plasmacytoid dendritic cells	Lin^- $HLA\text{-}DR^+$ $CD11c^-$ $CD123^+$ $BDCA2^+$	Leucocytes
Myeloid dendritic cells	Lin^- $HLA\text{-}DR^+$ $CD11c^+$	Leucocytes
$CD16^+$ mDCs	Lin^- $HLA\text{-}DR^+$ $CD11c^+$ $CD16^+$ $BDCA3^-$	mDCs
MDC1	Lin^- $HLA\text{-}DR^+$ $CD11c^+$ $CD16^-$ $BDCA3^-$	mDCs
MDC2	Lin^- $HLA\text{-}DR^+$ $CD11c^+$ $CD16^-$ $BDCA3^+$	mDCs
Tregs		
Regulatory T cells	$CD3^+$ $CD4^+$ $CD25^{high}$ $CD127^{low}$	CD4+ cells
Naive Tregs	$CD3^+$ $CD4^+$ $CD25^{high}$ $CD127^{low}$ $CD45RA^+$	CD4+ cells
nTreg	$CD3^+$ $CD4^+$ $CD25^{high}$ $FoxP3^+$ $Hellios^+$ $CD39^+$	CD4+ cells
iTreg	$CD3^+$ $CD4^+$ $CD25^{high}$ $FoxP3^+$ $Hellios^-$ $CD39^+$	CD4+ cells
Memory Tregs	$CD3^+$ $CD4^+$ $CD25^{high}$ $CD127^{low}$ $CD45RA^-$	CD4+ cells

Table S2. Immune cell profiles and their relationship with disease progression and MRD status prior to ASCT

Immune cell subsets prior to ASCT	Prognosis		*P*	MRD status prior to ASCT		*P*
	Non-progressive (n=28)	Progressive (n=5)		Negative (n=9)	Positive (n=24)	
Classical Monocytes	72.008 ± 12.583	64.149 ± 17.022	0.239	60.568 ± 17.393	74.212 ± 9.978	0.033*
Intermediate Monocytes	18.755 ± 9.885	26.253 ± 15.046	0.182	28.610 ± 15.539	17.305 ± 7.705	0.037*
Lymphocytes	0.909 ± 0.464	1.106 ± 0.602	0.408	0.627 ± 0.453	1.203 ± 0.432	0.009**
$CD3^+$ T cells	0.700 ± 0.359	0.793 ± 0.347	0.569	0.439 ± 0.270	0.918 ± 0.257	0.001**
$CD4^+$ T cells	0.323 ± 0.171	0.386 ± 0.251	0.501	0.186 ± 0.125	0.446 ± 0.174	0.002**
$CD8^+$ T cells	0.346±0.214	0.377±0.151	0.727	0.236±0.169	0.433±0.163	0.016*
DN T cells	0.025 ± 0.016	0.025 ± 0.015	0.997	0.015 ± 0.014	0.032 ± 0.012	0.008**
DP T cells	0.700±0.359	0.793±0.347	0.569	0.439±0.270	0.918±0.257	0.001**
$CD19^+$ B cells	6.256 ± 7.978	10.223 ± 11.278	0.356	5.296 ± 9.836	9.288 ± 9.018	0.353
Class-switched memory B cells	13.196 ± 10.903	8.586 ± 14.466	0.416	19.415 (2.255-31.175)	4.390 (2.460-10.380)	0.232
NK cells	15.262 ± 5.949	13.814 ± 5.953	0.594	19.093 ± 5.826	12.013 ± 4.054	0.004**
$CD16^+$ $CD56^{high}$ NK cells	0.660 (0.260-1.970)	1.795 (0.287-4.645)	0.374	0.240 (0.122-0.502)	1.970 (0.660-3.790)	0.006**
T4N	14.544 ± 9.140	13.241 ± 14.699	0.804	7.174 ± 6.199	18.278 ± 11.704	0.024*
T4CM	0.193 ± 0.104	0.213 ± 0.138	0.718	0.110 ± 0.086	0.257 ± 0.094	0.002**
T4E	0.005 ± 0.004	0.004 ± 0.006	0.822	0.002 ± 0.003	0.007 ± 0.005	0.032*
T4CM $CD28^+$ $CD27^+$ cells	92.680 (90.900-94.670)	91.845 (88.870-93.168)	0.336	93.140 (90.185-94.213)	91.270 (90.270-93.060)	0.595
T4CM $CD28^+$ $CD27^-$ cells	6.363 ± 3.000	9.178 ± 3.277	0.058	6.342 ± 4.203	8.108 ± 2.635	0.249
$PD1^+$T4CM	28.126 ± 12.354	48.966 ± 22.905	0.044*	29.893±9.672	42.482 ± 28.314	0.330
$CD8^+$ $CD57^+$ T cells	0.122 ± 0.079	0.138 ± 0.066	0.633	0.079 ± 0.060	0.158 ± 0.065	0.011*
T8N cells	12.070 (7.000-23.200)	4.640 (2.390-5.750)	0.029*	16.940 (8.090-26.070)	5.865 (4.768-6.835)	0.002**
T8CM cells	0.033 ± 0.023	0.034 ± 0.018	0.915	0.022 ± 0.020	0.041 ± 0.017	0.030*
$PD1^+$T8CM cells	0.013 ± 0.010	0.016 ± 0.006	0.442	0.008 ± 0.006	0.018 ± 0.008	0.008**
T8N $CD28^+$ $CD27^+$ cells	95.944 ± 2.787	88.514 ± 8.544	0.030*	93.120 ± 2.608	96.200 ± 1.185	0.003**
T8CM $CD28^+$ $CD27^-$ cells	4.983 ± 2.894	2.712 ± 1.104	0.048*	4.660 (3.027-8.227)	3.300 (2.590-3.730)	0.121
$CD8^+$ HLA-DR^+ T cells	68.392 ± 13.164	84.016 ± 11.822	0.048*	71.508 ± 13.292	72.260 ± 18.908	0.940
T8EM PD1+	55.320 (39.840-68.810)	57.515 (30.012-61.700)	0.547	54.079 ± 16.609	49.918 ± 17.110	0.591
T8E $CD28^-$ $CD27^-$ cells	0.089 ± 0.075	0.094 ± 0.054	0.853	0.044 (0.019-0.061)	0.104 (0.063-0.177)	0.016*

PD1^{+}T8E cells	22.649 ± 13.300	24.867 ± 14.882	0.726	30.609 ± 15.758	19.116 ± 10.429	0.058
Tregs	0.029 ± 0.019	0.029 ± 0.026	0.964	0.012 ± 0.010	0.040 ± 0.019	0.001**
Memory Tregs	0.025 ± 0.018	0.027 ± 0.024	0.831	0.009 ± 0.009	0.037 ± 0.017	0.001**
CD39+ Helios+ Tregs	33.408 ± 25.379	14.242 ± 20.951	0.090	35.039 ± 27.820	20.610 ± 22.679	0.209
CD39^{+} Tregs	0.015 ± 0.010	0.010 ± 0.013	0.368	0.007 ± 0.007	0.017 ± 0.012	0.044*
Foxp3^{+} Helios^{+} Tregs	40.467 ± 25.074	28.793 ± 29.542	0.345	44.814 ± 25.925	30.608 ± 26.808	0.247
pDCs	0.070 (0.050-0.080)	0.120 (0.110-0.155)	0.010**	0.090 (0.062-0.150)	0.090 (0.080-0.120)	0.913
T8N+T8CM /T8EM+T8E ratio	0.347 ± 0.194	0.278 ± 0.237	0.475	0.160 (0.108-0.229)	0.318 (0.240-0.540)	0.010**
CD8+PD1+/ CD4+PD1+ ratio	1.837 ± 1.166	1.444 ± 0.648	0.396	1.286 (1.182-2.038)	1.528 (0.900-2.163)	0.595

Note: Data are presented as mean ± standard deviation or median (interquartile range, IQR). All variables are expressed as percentages (%) unless otherwise indicated; absolute counts are expressed as $\times 10^9$/L. * $P < 0.05$, ** $P < 0.01$. ASCT, autologous stem cell transplantation; MRD, minimal residual disease; DNTs, CD4/CD8 double-negative T cells; pDCs, plasmacytoid dendritic cells; T4CM, CD4+ central memory T cells; T8CM, CD8+ central memory T cells; T8E, CD8+ effector T cells.

Table S3. Immune cell profiles and their relationship with disease progression and MRD status post-ASCT

Immune cell subsets post-ASCT	Prognosis		*P*	MRD status post-ASCT		*P*
	Non-progressive (n=28)	Progressive (n=5)		Negative (n=24)	Positive (n=9)	
Granulocytes	46.776 ± 10.376	55.924 ± 22.530	0.291	44.381 ± 10.883	63.998 ± 19.213	0.030*
Lymphocytes	40.150 ± 12.252	31.456 ± 19.365	0.303	42.426 ± 11.987	22.210 ± 13.737	0.018*
Lymphocytes	1.987 ± 1.649	1.119 ± 0.734	0.288	1.505 (1.205-2.095)	1.020 (0.771-1.040)	0.014*
transitional B cells	16.106 ± 8.103	28.606 ± 6.481	0.010 *	19.594 ± 10.284	24.367 ± 7.397	0.419
marginal zone B cells	2.800 ± 0.760	1.586 ± 1.428	0.048 *	2.661 ± 0.930	1.390 ± 1.137	0.050*
$CD16^{+}$ $CD56^{high}$ NK cells	3.855 ± 4.755	3.786 ± 3.762	0.978	0.945 (0.665-1.805)	7.285 (3.865-10.928)	0.024*
$CD3^{+}$ T cells	1.533 ± 1.261	0.917 ± 0.701	0.333	1.225 (1.030-1.913)	0.744 (0.471-0.882)	0.014*
$CD3^{+}$ HLA-DR^{+} cells	1.371 ± 1.228	0.833 ± 0.716	0.386	1.110 (0.888-1.770)	0.578 (0.333-0.740)	0.008* *
$CD4^{+}$ T cells	21.866 ± 12.176	28.626 ± 14.436	0.357	18.865 (15.502-21.802)	29.880 (21.968-39.812)	0.047*
$CD4^{+}$ HLA-DR^{+} cells	59.667 ± 10.372	76.610 ± 17.384	0.033 *	65.642 ± 15.168	67.875 ± 16.867	0.813
$PD1^{+}$T4E cells	41.312 ± 24.464	46.898 ± 34.775	0.722	54.927 ± 23.164	24.585 ± 17.040	0.037*
T4EM cells	42.778 ± 6.859	59.152 ± 15.792	0.013 *	48.818 ± 11.397	49.940 ± 17.990	0.889
T4CM cells	53.315 ± 8.565	38.204 ± 14.839	0.025 *	47.048 ± 11.512	47.657 ± 17.072	0.939
T4CM $CD28^{+}$ $CD27^{-}$ cells	0.016 ± 0.008	0.006 ± 0.003	0.022 *	0.014 ± 0.009	0.008 ± 0.006	0.209
$CD8^{+}$ T cells	73.017 ± 14.361	67.110 ± 13.225	0.456	77.196 ± 7.056	64.575 ± 11.716	0.027*
$CD8^{+}$ T cells	1.193 ± 1.163	0.654 ± 0.624	0.356	0.994 (0.736-1.400)	0.416 (0.239-0.564)	0.019*
T8 $CD28^{+}$ $CD27^{+}$ cells	0.493 ± 0.847	0.181 ± 0.194	0.437	0.280 (0.189-0.419)	0.100 (0.075-0.113)	0.024*
T8 $CD28^{-}$ $CD27^{+}$ cells	0.229 ± 0.208	0.143 ± 0.179	0.446	0.174 (0.142-0.351)	0.060 (0.026-0.104)	0.036*
$PD1^{+}CD8^{+}$ cells	0.715 ± 0.984	0.326 ± 0.298	0.410	0.520 (0.443-0.703)	0.167 (0.128-0.171)	0.002* *
$CD8^{+}$ HLA-DR^{+} cells	1.146 ± 1.135	0.632 ± 0.626	0.368	0.964 (0.715-1.350)	0.377 (0.219-0.511)	0.014*
T8EM $CD28^{-}CD27^{-}$ cells	35.709 ± 13.534	44.704 ± 11.293	0.225	34.705 ± 12.886	50.180 ± 8.426	0.049*
T8EM $CD28^{-}CD27^{+}$ cells	24.113 ± 5.313	19.086 ± 7.483	0.154	24.784 ± 5.273	17.053 ± 6.764	0.040*
T8EM $CD28^{+}$ $CD27^{+}$ cells	0.380 ± 0.707	0.125 ± 0.152	0.446	0.200 (0.137-0.328)	0.049 (0.034-0.065)	0.024*

PD1$^+$T8EM cells	60.358 ± 16.534	51.304 ± 23.663	0.400	63.392 ± 14.259	36.742 ± 14.164	0.008**
T8CM CD28$^+$ CD27$^-$ cells	0.002 (0.002-0.003)	0.001 (0.000-0.001)	0.040*	0.002 (0.001-0.003)	0.001 (0.000-0.001)	0.142
CD3$^+$ Vd1$^+$ cells	0.037 ± 0.025	0.024 ± 0.022	0.326	0.039 ± 0.023	0.011 ± 0.011	0.042*
CD3$^+$ Vd1$^-$Vd2$^-$ cells	0.029 ± 0.055	0.038 ± 0.050	0.771	0.014 (0.008-0.072)	0.002 (0.001-0.003)	0.009**
TCRgd$^+$ Vd1$^+$ HLA-DR$^+$ cells	88.285 (77.562-90.353)	88.180 (53.230-98.840)	1.000	86.762 ± 9.950	66.995 ± 23.468	0.041*
TCRgd$^+$Vd1$^-$Vd2$^-$ HLA-DR$^+$ cells	82.595 ± 15.842	78.404 ± 28.538	0.716	88.591 ± 12.584	61.395 ± 25.862	0.019*
CD3$^+$ TCR ab$^+$ cells	1.459 ± 1.258	0.852 ± 0.647	0.335	1.190 (0.943-1.740)	0.725 (0.464-0.850)	0.036*
CD3$^+$ TCR gd$^+$ cells	0.073 ± 0.076	0.065 ± 0.071	0.845	0.052 (0.043-0.117)	0.019 (0.007-0.032)	0.036*
TCR ab$^+$ CD8$^+$ T cells	0.970 ± 1.069	0.494 ± 0.570	0.374	0.798 (0.529-1.125)	0.234 (0.121-0.366)	0.014*
TCR gd$^+$ CD8$^+$ T cells	0.508 ± 0.556	0.268 ± 0.231	0.379	0.439 (0.328-0.571)	0.092 (0.052-0.164)	0.004**
TCRab$^+$ HLA-DR$^+$ cells	1.304 ± 1.228	0.772 ± 0.660	0.387	1.058 (0.828-1.548)	0.568 (0.328-0.722)	0.008**
TCRgd$^+$ HLA-DR$^+$ cells	0.065 ± 0.072	0.059 ± 0.071	0.879	0.049 (0.035-0.115)	0.010 (0.005-0.019)	0.008**
TN+TCM/TEM+TE ratio	0.489 ± 0.085	0.338 ± 0.176	0.040*	0.441 ± 0.169	0.416 ± 0.119	0.751
CD8$^+$PD1$^+$/CD4$^+$PD1$^+$ ratio	4.144 ± 2.342	2.636 ± 2.129	0.248	4.197 (3.438-5.245)	1.700 (1.264-2.082)	0.004**

Note: Data are presented as mean ± standard deviation or median (interquartile range, IQR). All variables are expressed as percentages (%) unless otherwise indicated; absolute counts are expressed as $\times 10^9$/L. * $P < 0.05$, ** $P < 0.01$. ASCT, autologous stem cell transplantation; MRD, minimal residual disease; T4CM, CD4+ central memory T cells; T8EM, CD8+ effector memory T cells; TN, naive T cells; TCM, central memory T cells; TEM, effector memory T cells; TE, effector T cells.
